# Associating protein residues in the literature with structural data

**DOI:** 10.1107/S2059798326000021

**Published:** 2026-01-26

**Authors:** Melanie Vollmar, Simon Westrip, Sreenath Nair, Balakumaran Balasubramaniyan, Sameer Velankar, Louise Jones, Peter Strickland

**Affiliations:** a Protein Data Bank in Europe European Molecular Biology Laboratory, European Bioinformatics Institute (EMBL–EBI) Wellcome Genome Campus, Hinxton CambridgeCB10 1SD United Kingdom; b International Union of Crystallography 5 Abbey Square ChesterCH1 2HU United Kingdom; University of Cambridge, United Kingdom

**Keywords:** text mining, large language models, text annotations, knowledge linking, protein structure

## Abstract

Protein structures are crucial in understanding the function, mechanism and disease-causing variants of proteins within any living cell. A number of experimental techniques are employed by researchers to determine such structures. Through structure inspection in molecular viewers, combined with supporting biochemical and biophysical experiments, scientists are able to identify the function of a protein, its reaction mechanism and effects caused by sequence variation. These detailed findings, supported by experimental results, are documented by being described in the scientific literature and by making the accompanying data open source. However, it has become increasingly difficult for a reader, in particular a non-expert, to access the correct additional information and assess the validity of the conclusions drawn based on experimental results. A reader is often required to resort to a number of different software packages to access the different data types. Here, we present a first-of-its-kind implementation of an artificial intelligence- and text-mining-supported software tool that allows the association of mentions in the text of one or more specific protein residues with their corresponding counterparts in the respective protein structure or structures. Our application allows a researcher to explore a residue of interest in the context of a publication and its respective protein structure, supported by its experimental evidence, in a single view. We describe model implementation, annotation extraction, downstream processing, dissemination and visualization at the IUCr and PDBe. The application presented is primarily aimed at readers of IUCr publications and users visiting the PDBe entry pages. However, we believe that in the future our application will be a valuable tool for reviewers of new submissions to IUCr journals and may even be useful as a curation tool involving the authors of a publication as annotation validators.

## Abstract 2

A software tool is introduced for associating text mentions of protein residues with their respective residues in a protein structure, with display in a molecular viewer.

## 1. Introduction

Writing scientific publications is a well established practice in research to document and disseminate novel findings, and provide background and details of the research undertaken. Written documentation ensures that findings are preserved for future reference and additional investigation. Documenting experimental details also enables other researchers to cross-examine and even reproduce experiments.

In structural biology, scientific publications focus on the mechanistic understanding of macromolecular function and describe residue-level, atomic details identified through the analysis of a protein structure. The experimental techniques employed to determine a protein structure, such as cryo-electron microscopy (cryoEM), nuclear magnetic resonance (NMR) spectroscopy or X-ray diffraction, can vary, and the findings suffer from different types of uncertainties and errors. However, the analysis and interpretation of protein structures all follow a similar process.

(i) Setting the scene: describe a structure’s overall architecture, secondary-structure elements, domain boundaries, motifs and other details from the existing literature.
(ii) Description of the experiment quality: analyse a structure in isolation and evaluate its quality based on established metrics.
(iii) Identify novel findings: compare a structure with related models from other organisms or in a different state to gain mechanistic and functional insights supported by non-structural biochemical experiments, often in combination with mutation studies.

To facilitate comparative analysis and gain novel insights from a protein structure, a researcher must familiarize themselves with the relevant literature and also consider deriving conclusions that take into account the experimental setup, data quality and results. The wwPDB (Berman *et al.*, 2003 ▸) provides a validation report at the time of structure deposition in the core PDB (wwPDB Consortium, 2019 ▸) or EMDB (wwPDB Consortium, 2024 ▸) structure archives. The validation reports are tailored specifically for each experimental technique used in structure determination, whether X-ray crystallography, cryoEM or NMR, and were designed by community experts as part of validation task forces (Read *et al.*, 2011 ▸; Henderson *et al.*, 2012 ▸; Montelione *et al.*, 2013 ▸; Baskaran *et al.*, 2024 ▸). These reports provide valuable information on the overall quality of the experimental data and the structure model, as well as detailed quality information on a per-residue basis. Expert users can view these validation data using specialized 3D molecule viewers. Knowledge and findings about the structure are accessed through scientific publications, which can be viewed via web-based browsers or require a PDF viewer to display the manuscript. Providing easy access to experimental evidence at the time when publications are accessed on a journal website can facilitate the interpretation of biological insights presented in the publication for both structural and non-structural biologists, and support the use of structure data to drive life-science research. It is especially important to ensure that mentions of specific residues with functional roles are supported by experimental evidence, thereby allowing confident planning of further experimental studies using this information.

Named entity recognition (NER) is the process of identifying words and phrases of interest in a text and labelling the found text spans according to a pre-defined set of entity type classes. For example, in the sentence ‘A car drives along the road’ the term ‘car’ represents a named entity of type ‘vehicle’, if this is the label chosen for that named entity. In the particular application presented here, our interest lies with identifying key information about specific and often functionally critical residues. Traditionally, text mining relied on rule-based approaches to identify relevant knowledge as named entities in a text. These rules were hand-crafted and highly specific to a particular application, and relied on the deep expertise of domain experts. However, all rule-based approaches lacked the aspect of contextualizing the found named entities.

Named entity linking involves linking a found named entity to a reference resource such as a controlled vocabulary or an ontology to disambiguate the found text span. Controlled vocabularies and ontologies are reference sources that provide relationships between concepts or entity types, often in a hierarchical structure, with detailed definitions and assign unique identifiers to these concepts. For our purpose of working with protein structures, UniProt (The UniProt Consortium, 2025 ▸), via the SIFTS-mapping process (Structure Integration with Function, Taxonomy, and Sequence; Dana *et al.*, 2019 ▸), was the reference source of choice. By linking a found term through a unique concept identifier to a reference source, text spans and their meaning within their context can be disambiguated. For example, in the sentence ‘The bank was located along the bank of the river’ the term ‘bank’ can be interpreted as a financial institution (‘financial’ as the entity type label) or the edge of a river (‘geographical’ as the entity type label) depending on its context. As a financial institution the term will be linked to a financial ontology, whereas for the geographical meaning we link the named entity to a geo­graphical ontology. In the case of protein structures, disam­biguation ensures that the identified protein residue in its written context is associated with its correct counterpart in the reference sequence and structure to provide sequence, chemical and biological context.

In the past, attempts were made to identify mentions of functionally important residues in the literature using rule-based text-mining approaches, such as in *pyresid* (Firth *et al.*, 2019 ▸), *mutation grounder* (Laurila *et al.*, 2010 ▸; Klein *et al.*, 2012 ▸), *MutationFinder* (Caporaso *et al.*, 2007 ▸) or *Open Mutation Miner* (*OMM*; Naderi & Witte, 2012 ▸). These systems all adhere to a similar sequential execution of entity extraction and generation of candidate lists of named entities, followed by filtering and selecting the best-fitting candidates based on expert-defined criteria. *pyresid* is an application co-developed by EMBL–EBI and the Science, Technology and Facilities Council (STFC) aimed at identifying mentions of amino acids in publications and linking them to their respective protein. *Mutation grounder* was developed to identify amino-acid mutations in publications and link them to reference sequence positions and accessions in UniProt (The UniProt Consortium, 2025 ▸). Graph-association rules were used by Ravikumar *et al.* (2012 ▸), allowing the contextualization of residue–protein pairs and encoding their relationship across longer distances beyond sentence boundaries. Although these systems represent initial attempts at curating unstructured information from the scientific literature into structured knowledge, they were all limited by the fine-grainedness of the decision rules that could be crafted. None of these systems was context aware and they were prone to language-ambiguity-related misidentification of relevant details.

The rapid developments in the field of natural language processing (NLP) and text mining, in particular applications for transformer and (large) language models (LLMs), enabled the processing of large amounts of unstructured scientific literature. Unlike a keyword search or the rule-based approaches, which identify text spans as perfect matches regardless of their contextualized meaning, these recent developments in NLP allow the identification of named entities within the publication text based on context. In the case of the ‘bank’ example above, the two different meanings will each have a unique concept identifier from their respective reference source linked to their entity type label. The surrounding context informs the text-mining algorithm when to apply the ‘financial’ or the ‘geographical’ label.

Thus, identifying, labelling and extracting named entities and their surrounding context is the first step in structuring knowledge. Furthermore, organizing the contextualized extracted knowledge in open-access databases supported by experimental evidence alongside visualization of protein models allows dissemination to a wide range of scientific communities.

We present here the details of a novel application that associates residue mentions in publications in the IUCr journals, *Acta Crystallographica Section D*, *Acta Crystallographica Section F* and *IUCrJ* in particular, with their respective residues in PDB structures, alongside data-quality statistics from experimental results. The application uses a fine-tuned transformer model described in Vollmar *et al.* (2024 ▸) to perform named entity recognition to identify any mentions of a protein residue in the format amino-acid name and sequence position, as well as point mutations. Amino acids can be identified in both three- and single-letter notation. Downstream pipelines utilize SIFTS mapping (Dana *et al.*, 2019 ▸) to map UniProt reference sequences and validation files provided by PDBe (Armstrong *et al.*, 2020 ▸) for each structure linked to a publication to pair the residue identified in the literature with its equivalent in the structure and its corresponding reference sequence in UniProt.

Our application is currently intended as a reading support for users studying scientific publications in IUCr journals through an online browser. For the PDBe entry pages our goal is to enrich the structures with written context and scientific findings. The readers or users are not expected to be structural biology experts, but rather scientists who want to understand proteins and their functions for their particular field of research. By connecting the text mentions, reference sequence, experimental evidence and molecule display, we hope to enable any researcher to evaluate findings in an informed manner. Furthermore, the harvested residue–structure–publication associations can also be accessed through application programming interfaces (APIs) at PDBe, which enables those from a computational field to use annotations in computational work.

In future implementations, we envisage placing the tool within the review pipeline at the IUCr to make it available to reviewers and editors at the point of new publication submissions. It is hoped that an inline association between text mentions, structure display and experimental statistics will support reviewers and accelerate the review process. Another potential future extension could be to not only focus on individual residues and point mutations but also provide grounding and linking of additional text mentions, for example, a protein’s name, protein family, functional state and cellular location. Our underlying predictive model already identifies additional terms. However, we found that existing reference sources, such as ontologies and controlled vocabularies, often cover only a portion of the required terms. The lack of reference terms can be attributed to the fact that ontologies are often created as a part of a specific research project and, in many cases, are not added to larger ontologies with which they overlap or to ontology repositories such as the Ontology Lookup Service (OLS; McLaughlin *et al.*, 2025 ▸). The ontology most suitable for describing protein structure features is the Sequence Ontology (SO; Eilbeck *et al.*, 2005 ▸). However, to comprehensively expand the term coverage in SO is beyond the scope of the current work. One possibility to increase the coverage in the longer term could be to create annotations as part of the review process and have the authors validate the proposed annotations before handing them to the most suitable ontologies for integration.

## 2. Materials and methods

### 2.1. Implementation

The transformer model, v2.1, underpinning the NER step has been described previously (Vollmar *et al.*, 2024 ▸) and can be freely accessed and downloaded from https://huggingface.co/PDBEurope. The base model, PubmedBERT (BERT: Bi­directional Encoder Representations from Transformers; Gu *et al.*, 2021 ▸), pre-trained on the whole scientific literature content of PubMed, was fine-tuned to carry out NER for structure-specific terminology. Because it is pre-trained on bio­medical literature, PubmedBERT understands how bio­medical scientific literature is written and has learned scientific terminology as part of its word embeddings, *i.e.* it captures the semantics of biomedical literature, entities and concepts. The pre-trained PubmedBERT can be fine-tuned and optimized for different applications such as text summarization, question answering, document classification and named entity recognition. It is not a large language model, and one cannot interact with it through instructions and prompts.

20 different entity types [bond interaction, chemical, complex assembly, evidence, experimental method, gene, mutant, oligomeric state, protein, protein state, protein type, ptm (post-translational modification), residue name, residue number, residue name number, residue range, site, species, structure element and taxonomy domain; please see the supplemental material to Vollmar *et al.* (2024 ▸) for further details regarding the different entity types] are recognized by our model with differing accuracies. The two entity types used for developing the association pipeline are ‘residue name number’ with precision 0.95, recall 0.96 and F1 score 0.96, and ‘mutant’ with precision 0.91, recall 0.97 and F1 score 0.94. Here, precision is the ratio of correctly predicted positive samples (*i.e.* true positives) and all samples in the data set predicted to be positive (*i.e.* true positives and false positives): precision = true positives/(true positives + false positives). Recall is the ratio of correctly predicted positive samples and all positive samples in the data set (*i.e.* true positives and false negatives): recall = true positives/(true positives + false negatives). F1 score is the harmonic mean of model precision and recall: F1 score = 2 × (precision × recall)/(precision + recall). ‘residue name number’ is used to label text mentions of an amino acid with a sequence number and ‘mutant’ refers to a point mutation. The remaining 18 entity types are also collected but not processed. The codebase for the article annotation pipeline was developed in Python 3.9.12.

#### 2.1.1. Preparing the input text for prediction

Within the IUCr systems, publication text is available as JATS (Journal Article Tag Suite; see https://jats.nlm.nih.gov/versions.html for the current version) XML (Extensible Markup Language) files. An example file for a publication in IUCr JATS XML is provided in the code repository in the folder examples/IUCr_JATS_XML. Using XML tags such as <sec>, <sec-type> and <p>, we identified the raw text passages and section titles for each publication. The identified raw publication text was split into individual sentences. The sentences were collected and enriched with additional information such as a unique sentence identifier, a sentence character count start (which was always ‘0’), a sentence character count end (which was the total character count for a sentence) and the section that a sentence belonged to. The additional information enabled tracking of sentences in the annotation pipeline and matching the annotations to specific locations within a sentence. The sentence dictionary created was used for labelling.

#### 2.1.2. Preparing the model for labelling

To efficiently run labelling with the model described in Vollmar *et al.* (2024 ▸), we applied quantization to the original model, v2.1. During this quantization step, we used optimum[onnxruntime] 1.2.2 to convert a 32-bit floating-point model to an eight-bit integer model. Reducing the precision of the model also reduced its size from 1.3 GB to 105 MB. The smaller model could therefore be deployed on a CPU-only machine for inference. To assess the performance of the model after quantization, we performed the SemEval evaluation (Segura-Bedmar *et al.*, 2013 ▸). The evaluation employed an independent validation set used for benchmarking, which was published in Vollmar *et al.* (2024 ▸).

#### 2.1.3. Annotating with the model

The maximum number of tokens the model could process was 512. Therefore, we combined eight sentences into a batch and used a batch size of four (32 sentences) for labelling. For each sentence in a batch, a list of predicted named entities was returned, with each entity defined by a start and end character count. This information was used to locate the text span in the sentence. Each annotation was therefore defined by a start and end character count, the exact text span, the predicted entity type and a confidence score for attaching a particular class label. All annotations for a sentence were then added to the sentence dictionary created earlier.

#### 2.1.4. Harvesting annotations

The sentence dictionary followed the Europe PMC annotation format as described in their instructions for annotation submission (https://europepmc.org/AnnotationsSubmission). All annotations were harvested into a final JSON (JavaScript Object Notation) file, with each annotation being described by the keys below.

‘exact’: the exact text span covered by the annotation.
‘position’: the location of the annotation in a sentence, defined by the unique sentence identifier and the position of a word in the sentence, as given by the word count in the sentence, *e.g.* 25.18 refers to the 18th word in the 25th sentence.
‘prefix’: 30 characters in a sentence preceding the exact text span.
‘postfix’: 30 characters in a sentence following the exact text span.
‘type’: the predicted entity type for the annotation.
‘ai_score’: the confidence score of the algorithm for the predicted annotation.
‘char_start’: the starting position of the exact match in a sentence.
‘char_end’: the ending position of the exact match in a sentence.
‘tags’: for any additional details to support the annotation, for example references to ontologies or controlled vocabularies; here, we create the associations with the reference sequences in UniProt.

At this point, we also evaluated whether the model had annotated adjacent text spans as independent instances rather than a continuous annotation. For entities of the same type that were directly adjacent based on their character counts, we combined their annotations.

#### 2.1.5. Post-processing annotations

A number of post-processing steps were implemented to address errors in identifying text spans or assigning the correct entity type label.

A series of regular expression definitions were created to identify whether a found named entity followed expected patterns. For the entity type ‘residue name number’, the pattern consisted of a three-letter amino-acid name and an integer for the sequence position, *e.g.* Arg155. If the entity type was predicted to be ‘mutant’, then the pattern was a single-letter native-amino-acid name and sequence position, followed by another single-letter amino-acid name for the mutation, *e.g.* R155A. In cases where a single-letter amino acid was found, this was expanded to the corresponding three-letter amino acid, *e.g.* Arg155Ala. If a found text span did not fit any of these options, for example due to additional characters, we tried to identify the most probable text span that would match the pattern. Only text spans that could be resolved to follow any of our expected patterns were then subjected to residue matching for associated structures using the PDB validation XML files.

To fix incorrect entity type labels, we again relied on the regular expression patterns. If the pattern did not support the found entity type, we changed the label to the one that returned a regular expression match. For example, if Arg155 was labelled ‘mutant’ a search for the mutant pattern failed, whereas that for ‘residue name number’ succeeded. Consequently, the entity type label was changed from ‘mutant’ to ‘residue name number’.

Finally, all annotations were written to a Europe PMC standard JSON file.

#### 2.1.6. Enriching the sentence dictionary

Additionally, in line with the basic set of annotation requirements based on the Europe PMC standards, we added a set of optional information. These additional details covered article identifiers such as PubMed and PubMed Central identifiers, digital object identifiers (DOIs), IUCr-specific identifiers and the publishing licence. We also added details about PDB identifiers for structures linked to a publication, the known organism with synonyms, taxonomy identifiers, and UniProt accessions for the proteins found using the PDB identifiers. All information was retrieved through API calls either to Europe PMC using the DOI or title of an article, or to PDBe using the linked PDB identifier. The latter was possible because the IUCr records PDB identifiers for each publication, which can be easily retrieved through the XML tags <ext-link>, <ext-link-type> and <xlink:href>. ‘IUCr’ was added as a provider to keep track of who provided annotations, as they will be shared with PDBe and added to their database.

#### 2.1.7. Enriching the annotations

Following best practices in the text-mining and NLP field, the annotated residues of entity type ‘residue name number’ and ‘mutant’ were associated with UniProt references for disambiguation where possible. We used the SIFTS mapping files provided by PDBe, which allow a per-residue mapping between a residue in a protein structure and its equivalent residue in a UniProt accession. Furthermore, the goal of our application was also to provide readers with experimental details to support the analysis described in the text. We therefore used the per-residue validation details provided by the PDB in the validation XML files to extract relevant statistics for each annotated residue. We have provided examples of SIFTS and validation XML files in connection with the example for a publication in IUCr JATS XML mentioned in Section 2.1.1. The files are in the code repository for this publication in the directories examples/SIFTS_XML and examples/validation_XML.

The details found for the SIFTS mapping and the validation XML were collected and attached to the ‘tags’ key of the relevant annotations, *i.e.* those with entity type ‘residue name number’ or ‘mutant’. The following details were extracted for all annotations.

‘pdb_id’: the PDB identifier that the details belong to.
‘pdb_res_name’: the residue name in the structure, *i.e.* the amino-acid name.
‘pdb_res_number’: the author-provided residue number in a structure.
‘pdb_res_seq’: the residue number in a structure as counted from the first modelled residue.
‘pdb_res’: a combination of the residue name and the author-provided residue number in a structure.
‘pdb_chain’: the chain identifier in a structure where the residue is found.
‘ramachandran’: the Ramachandran score found for a residue.
‘rotamer’: the rotamer found for a residue side chain.
‘phi’: the phi angle found for a residue.
‘psi’: the psi angle found for a residue.
‘clashes’: a list of any clashes that have been found for a residue.
‘altconf’: any alternative conformations that have been found for a residue side chain.
‘wildtype_pdb_res’: the wild-type residue, if the entity type was ‘mutant’; otherwise this is empty.
‘uniprot_id’: the UniProt accession for the protein that the residue belongs to.
‘uniprot_name’: the UniProt accession and species extension for the protein that the residue belongs to.
‘uniprot_res’: a combination of the UniProt residue name and residue number in the sequence.
‘uniprot_uri’: the URI to the UniProt reference entry.

There were additional details that are specific to the experimental method used to determine the protein structure.

For a structure determined by an X-ray diffraction experiment, we additionally provided:

‘rscc’: the real-space correlation coefficient found for the residue.

If the structure was determined using cryo-electron microscopy, we additionally provided:

‘q_score’: the *Q*-score found for the residue.

In the case of an NMR experiment used for structure determination, we additionally provided:

‘distances’: a list of distance outliers found for the residue.
‘angles’: a list of angle outliers found for the residue.

#### 2.1.8. Highlighting annotations in the source article

In a web environment, such as viewing a publication in a browser on a journal’s website, the ‘prefix’ and ‘postfix’ content of an annotation are sufficient in most cases to identify the sentence containing the ‘exact’ text span covered by the annotation. This text-span identification follows the ‘Text Quote Selector’ approach as described in the World Wide Web Consortium (W3C) recommendation for a Web Annotation Data Model (https://www.w3.org/TR/annotation-model/).

Once annotations have been associated with an article, IUCr Journals use JavaScript (ECMAScript 2017) to retrieve the JSON data asynchronously and dynamically highlight the associated text in the HTML (Hypertext Markup Language) rendition of the article, including links to present a summary of the annotation data, as well as to display the associated structure in the molecular-visualization tool Mol* (Bittrich *et al.*, 2024 ▸; Midlik *et al.*, 2025 ▸), as described in Section 2.1.9.

#### 2.1.9. Visualizing residues in linked structures

Currently, IUCr Journals use the PDBe Molstar Plugin (imported from https://cdn.jsdelivr.net/npm/pdbe-molstar@3.2.0/build/pdbe-molstar-plugin.js) to display PDB structures in pop-up ‘widgets’. Highlighting a specific residue in the widget for a PDB structure is straightforward using the PDBe Molstar Plugin API. The residue can be selected using the ‘select’ method and specifying properties such as chain (*e.g.* struct_asym_id: ‘A’) and residue ID (*e.g.* residue_number: 100), along with various rendering options (*e.g.* representation: ‘ball-and-stick’, representationColor: {r:255,g:255,b:0}). Noncovalent interactions and volumes can be added to the focused region of the structure via a simple ‘interactivityFocus’ method. For further information about implementing the PDBe Molstar Plugin on web pages, please see https://github.com/molstar/pdbe-molstar/wiki.

#### 2.1.10. Sharing annotations with PDBe

Annotations were designed to be shared and reused across different hardware and software platforms as we adhere to the Europe PMC annotation JSON standard. Currently, annotation JSON files are being uploaded by IUCr Journals to a designated FTP (file-transfer protocol) area within the EBI infrastructure. From this location, a processing pipeline developed by the PDBe team quality-checks and loads the annotations into their relational database. A set of four API endpoints (see documentation at https://www.ebi.ac.uk/pdbe/aggregated-api/ under the sections PDB and UniProt) were developed to access the annotation information in the database. The endpoints fetch database content based on a PDB entry or UniProt accession with the option to specify a particular protein chain and residue. Furthermore, the annotations are displayed via the newly designed PDBe webpages, which provide direct access for browsing and exploring the information when navigating through a PDB entry. Details of the webpages will be published elsewhere.

### 2.2. Hardware requirements

The annotation software will run on a modest PC, for example 8× Core x86 CPU @ 1.00 GHz + 16 GiB RAM.

### 2.3. Code availability

The code for the annotation and validation pipeline developed for this application has been published in a Github repository and is fully open access: https://github.com/PDBeurope/IUCr-annotation-pipeline.git.

## 3. Results

### 3.1. Model quantization

The model quantization is a necessary step to improve the computation time during labelling as well as to reduce hardware requirements. However, this comes at the cost of reduced prediction performance. Table 1 ▸ gives the prediction confidence of the model, either in its full or quantized version, for an unseen sentence. The quantized model displays lower values by 0.01–0.02 for the prediction confidence depending on the entity type.

Additional assessments were carried out using the SemEval validation scheme. Tables 2 ▸, 3 ▸ and 4 ▸ show the differences in performance, measured by precision, recall and F1 score, between the full and quantized models on an unseen, independent validation set. Across this much larger benchmarking set, there is a loss of ∼0.05 for the different metrics for the quantized model compared with the full model. For the entity types ‘mutant’ and ‘residue name number’, which were the main focus of our pipeline, the performance changes are given in Tables 5 ▸ and 6 ▸, respectively. We find that the performance of the quantized model for ‘mutant’ drops by ∼0.1 for precision, recall and F1 score. This finding comes on the back of already poorer prediction performance for this entity type compared with ‘residue name number’ for the full model. For ‘residue name number’ the values for the precision, recall and F1 score are lower by 0.04–0.07, in line with the overall observed change, while the full model displays issues with overfitting, indicated by a recall of 1.

### 3.2. Post-processing annotations

It should be noted that although many checks have been implemented to correct errors in labelling and identifying entity types, some errors may still occur. The underlying reasons may be due to formatting errors in the JATS XML file from which the text was extracted so the text cannot be ‘read’ by the model, or the model itself may have missed a text span or assigned the wrong entity type labels. A text span may not have been encountered before, and the post-processing step does not contain the necessary regular expression pattern to fix the error.

An example of such an error was found in https://doi.org/10.1107/S205979832300311X (Miyagi *et al.*, 2023 ▸). Here, the algorithm correctly identified ‘Glu89H’ (‘exact’: ‘Glu89H’) as the relevant entity in the sentence ‘Although Glu89H is involved in both salt bridges, the basic counter-residues are not conserved; this is because complexes A and B are nearly, but not perfectly, in twofold symmetry’. The algorithm correctly identified the entity type, ‘type’: ‘residue name number’, and the annotation had very high confidence, ‘ai_score’: ’0.9989009’. However, the post-processing step failed to remove the additional ‘H’ in this case, and the annotation does not appear on the webpages. In the preceding sentence, the named entity ‘Glu89^H_complex*B*^’ is correctly processed, removing ‘H_complexB’ and associating with its reference sequence in UniProt, as well as being displayed in Mol*.

We also found that the model occasionally assigned the wrong entity type to an annotation. If an annotation was wrongly labelled ‘residue name number’ but was found to be of type ‘mutant’ through regular expression pattern matching, then we changed the entity type label accordingly and set the value for the key ‘annotator’ to ‘post_processing’ and the value for the key ‘ai_score’ to ‘removed’.

### 3.3. Enriching the sentence dictionary

Although the PDB has been curating protein structures for several decades, this is a semi-automated process for which requirements and standards have changed over time. In particular, associating a PDB entry with its relevant literature involves authors informing curators at the PDB that a publication has become available. This reliance on external information and authors’ delay in updating records result in missing structure–publication associations.

At the IUCr, publications are enriched by adding associations to the structures for which a publication provides a primary citation for a PDB entry. By extracting this additional information directly from the publication JATS XML, we were able to update 94 entries in the PDB with a primary citation from 35 publications; see also Section 3.5.

### 3.4. Enriching annotations and mapping to references

SIFTS enabled the mapping of residues in a PDB entry to their counterparts in a UniProt accession. It should be noted that the process of SIFTS mapping only worked successfully if the authors used either the residue numbering found in the PDB structure or the numbering in the UniProt accession to refer to the residues in their publication. During the mapping process, there were a few cases where the authors did not use either numbering scheme when referring to a residue in the text and therefore no link could be established. If a link was missing, we were also not able to further enrich an annotation with structure-quality metrics from the validation XML file available for a PDB entry.

### 3.5. IUCr Journals archive predictions

A total of 9152 publications from the journals *Acta Crystallo­graphica Section D*, *Acta Crystallographica Section F* and *IUCrJ* starting from the year 2000 were submitted to the labelling and enrichment pipeline. The year cutoff was chosen based on journal editor recommendation, but also on the fact that older articles have been digitized from hard copies, often as images, which make them inaccessible for text-processing models and pipelines as described here. Almost half of the publications (4124) had at least one structure linked. For the open-access publications, the annotations covered all 20 possible entity types, with those of type ‘residue name number’ or ‘mutant’ having undergone additional enrichment. If the publication was not open, then only annotations for ‘residue name number’ or ‘mutant’ were recorded. This latter restriction was due to licencing considerations, as extracting the context for all possible entity types would expose large parts of a publication’s content. In total, 3153 publications returned a JSON file with annotations; 936 publications did not return valid annotations, and 35 publications had not been recorded as a primary citation for a protein structure in the PDB, which was corrected (see Section 3.3) and annotations were added during a second round of pipeline execution. The successfully processed publications contributed a total of 781 892 rows of annotations to the PDBe relational database associated with just over 10 000 structures.

### 3.6. Accessing the annotations in a live document

**Figure 1:**
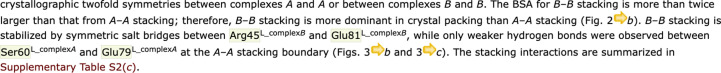
Example of highlighting residue mentions in a publication for which a link to a UniProt reference and to a residue in a structure were established.

**Figure 2:**
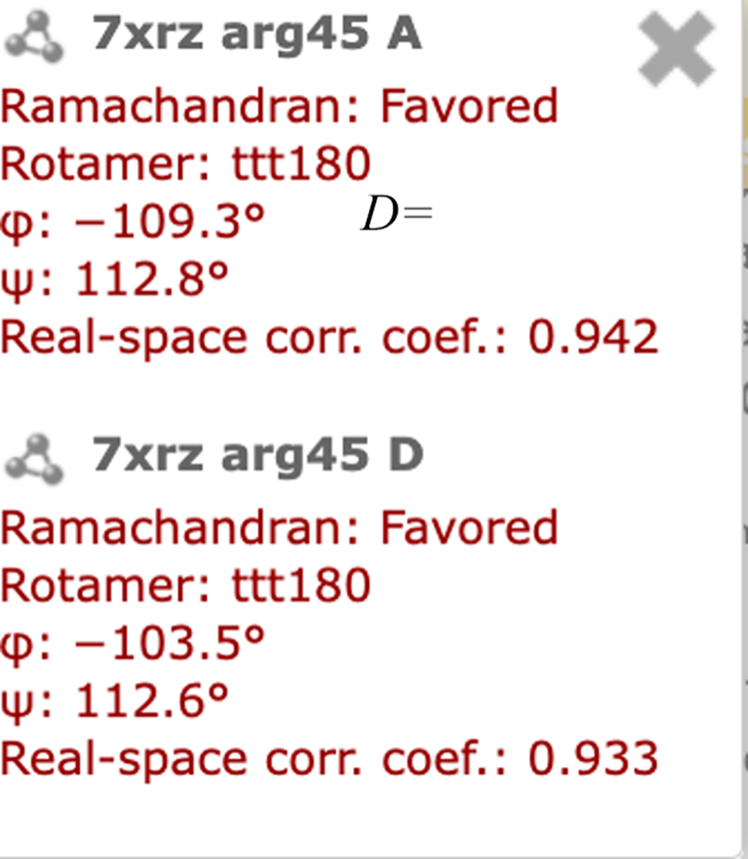
List of available structures containing the highlighted residue.

**Figure 3:**
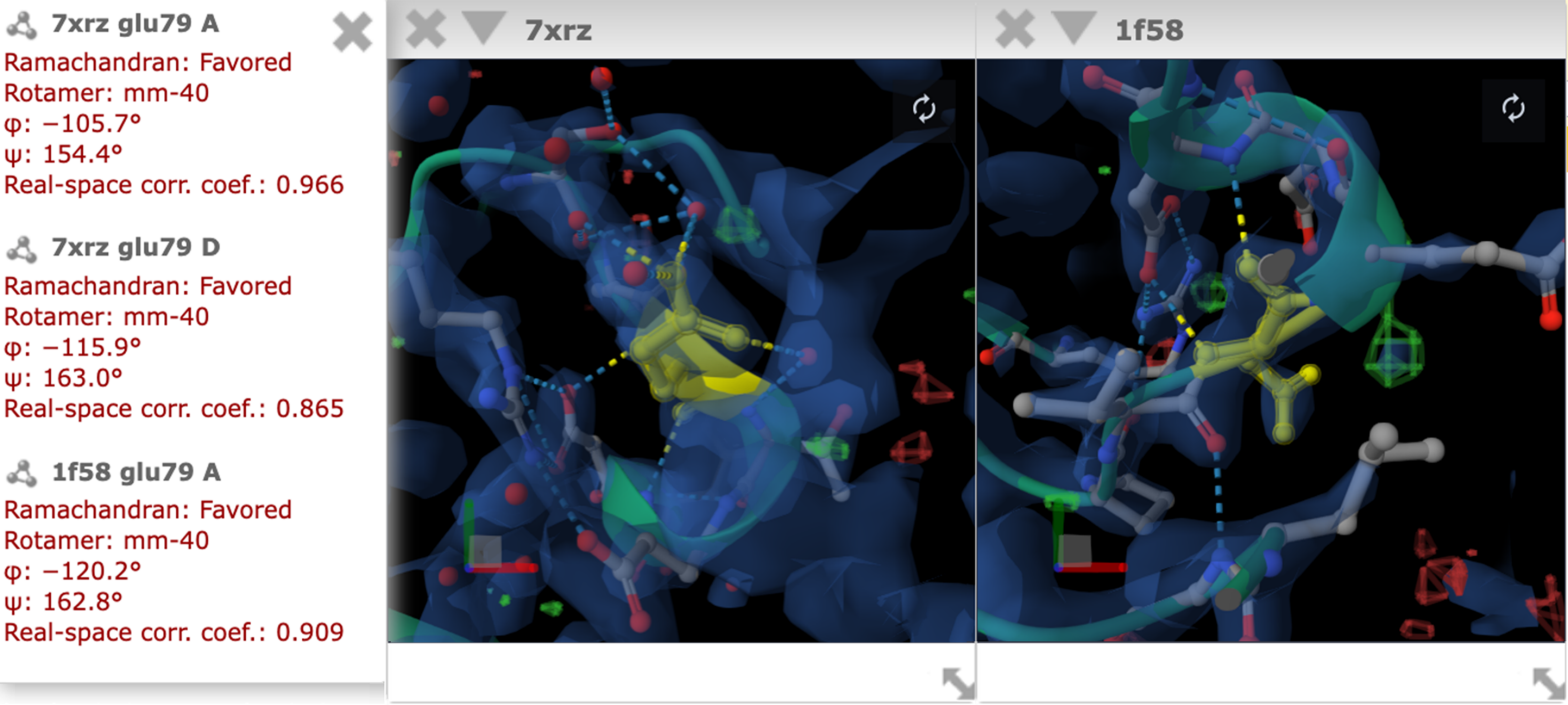
Example of how two different PDB entries can be displayed next to each other in separate Mol* windows.

**Figure 4:**
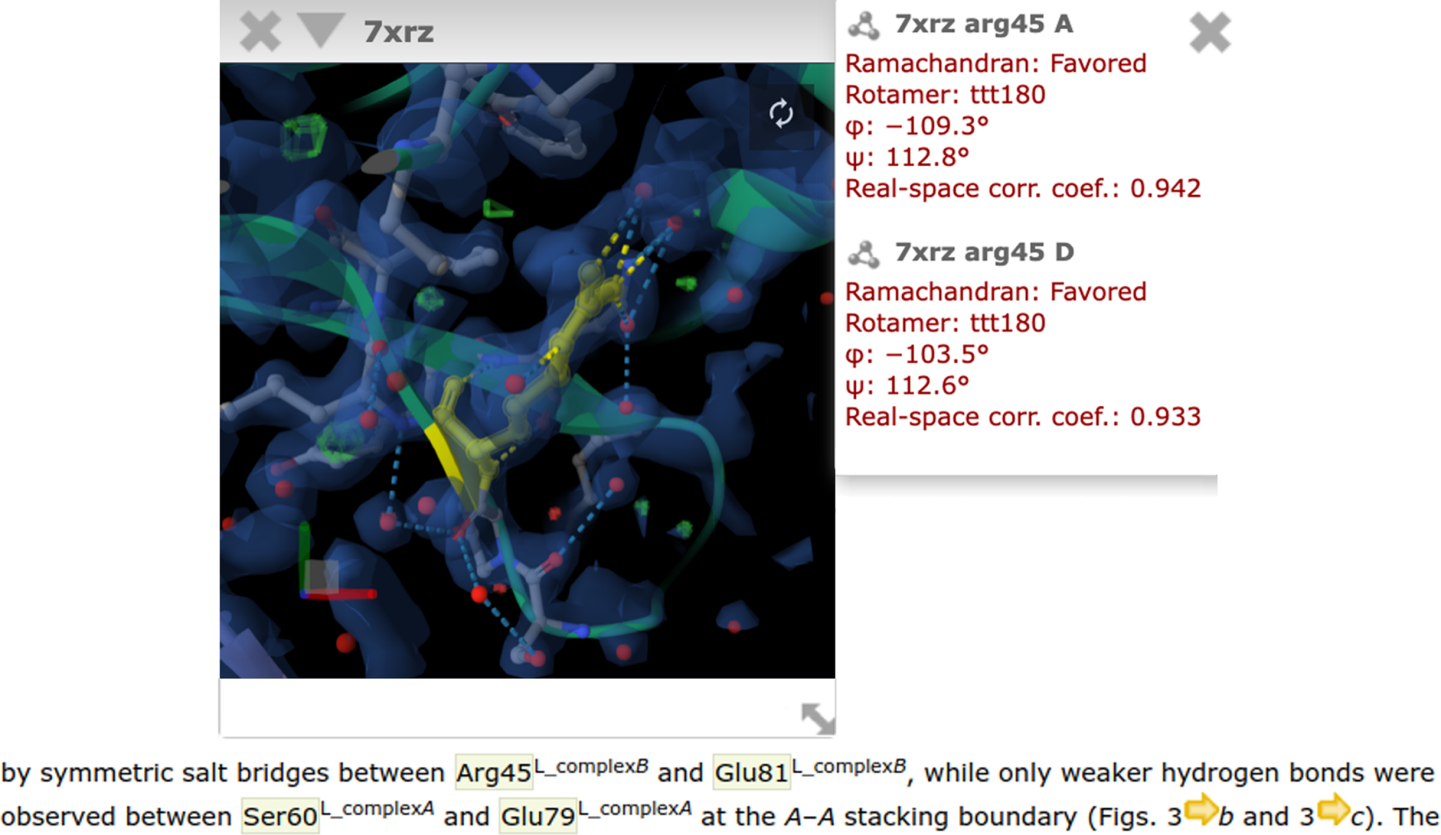
Example of how a highlighted residue is linked to a structure and displayed in the Mol* molecule viewer.

In a web environment, IUCr Journals provide a number of annotations and enrichments for their publications. The annotations that will be applied can be selected by the reader. If a publication in one of the IUCr journals contains mentions of a residue with a sequence number or a point mutation, these are highlighted in the text on the webpages (Fig. 1 ▸). By clicking on the highlighted residue, a selection panel will appear, which allows the selection of a structure (Fig. 2 ▸). All structures that contain the highlighted residue are listed. For each listed occurrence of a residue, quality metrics are provided. Four quality metrics are provided regardless of the experiment type: Ramachandran score, rotamer and phi and psi angles. For a structure determined in an X-ray diffraction experiment, we display the real-space correlation coefficient (RSCC) for the residue. In the case where a structure has been determined by a cryo-electron microscopy experiment we display the *Q*-score, and for an NMR experiment violations for distances and angles. RSCC, *Q*-score and violations to bond angles and distances are metrics of how well the atomic coordinates of a model fit to the data. They allow the reader to judge whether the structural background of a PDB entry supports the interpretation and statements by authors in a publication. On clicking on the PDB entry, residue and chain label in the list of associations, the Mol* structure viewer is launched in a detached, separate window overlaying the text. It can be moved and arranged freely. This is particularly useful if one wants to look at and compare different structures, as each PDB entry will be displayed in its own window (Fig. 3 ▸). Residues found in the same PDB entry are displayed in the same window, with the molecule centred on the selected residue. Once a molecule is displayed, it will be centred on the selected residue and chain and is displayed in cartoon style, with the specific residue appearing as a ball-and-stick model coloured yellow. Found interactions, such as hydrogen bonds and salt bridges, are displayed alongside the experimental data, if available, for the selected residue and its surrounding area. For a structure derived from an X-ray diffraction experiment, an electron-density map will be displayed. If cryo-electron microscopy was used to determine a protein structure, then an electric potential map is displayed. Fig. 4 ▸ shows an example of how the residue and molecule are displayed. If a residue appears in several chains, such as chains ‘A’ and ‘D’ in our example, then one can switch between the residues by clicking on the residue in another chain. This will not open a new window, but will centre the display on the selected residue instead. To support readers and showcase the benefits of an inline molecule viewer connected to a publication, we have recorded a tutorial video (Supplementary Video S1).

### 3.7. Accessing the annotations at PDBe

Annotations created by the annotation pipeline at the IUCr are uploaded to a dedicated FTP area at PDBe. As part of the PDBe weekly release pipeline, these annotations are ingested and added to the PDBe central database. Annotations are currently accessible in two ways: programmatically via the PDBe API endpoints or as part of the entry pages under the tab ‘Text Annotations (AI)’.

The API documentation allows exploratory work regarding the returned content and structure of information. Novice users are able to visualize the information to understand API functionality, and expert users can identify the exact keywords required to pick and mix the content they want to extract from the returned information. For API access, annotations can be grouped by PDB entry or UniProt accession with and without selecting a specific chain and/or residue. Annotations retrieved through the API are also not restricted to a single origin. Grouping by PDB entry or UniProt accession allows annotations from multiple IUCr publications to be combined.

**Figure 5:**
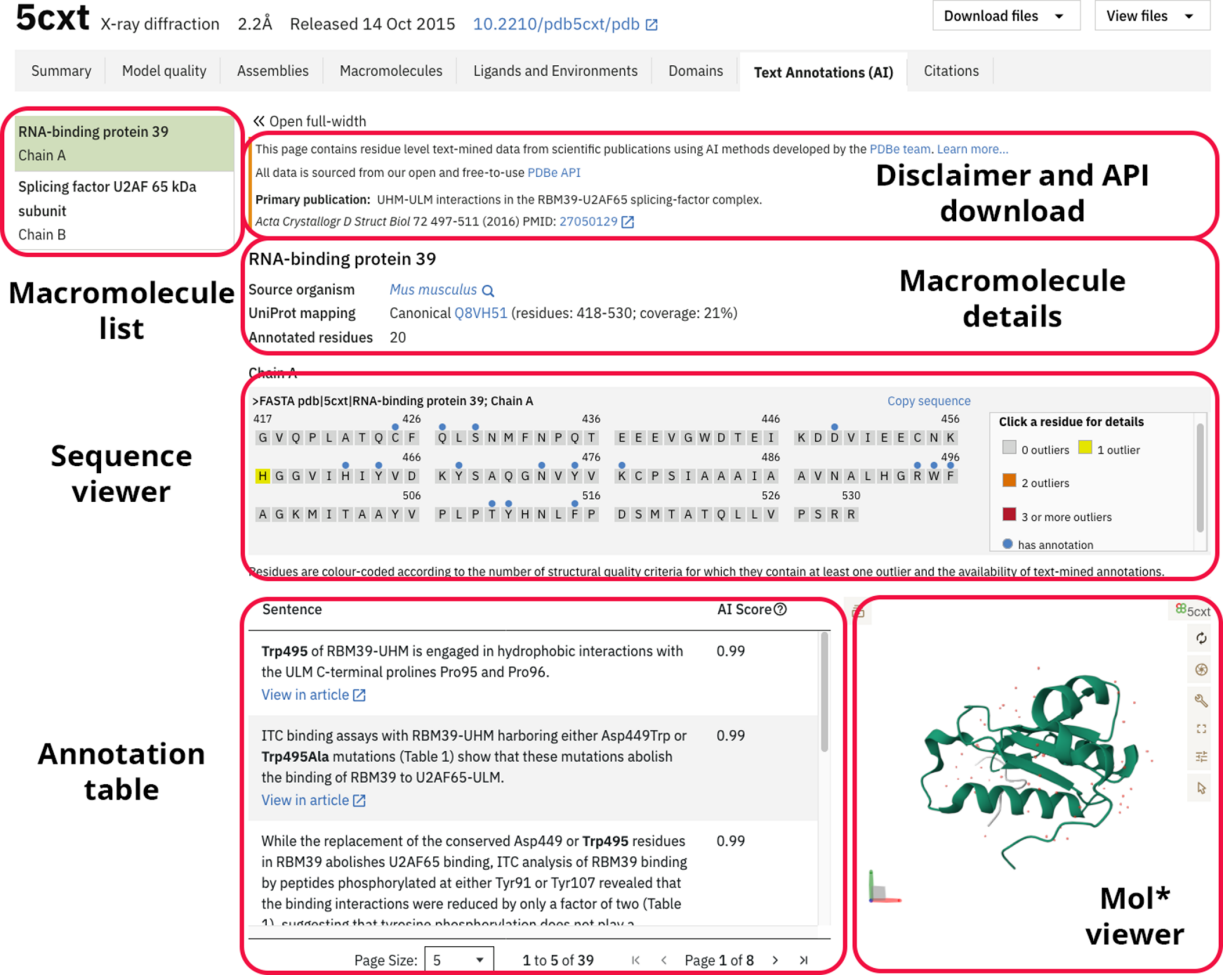
Annotation visualization on the PDBe entry pages for structure 5cxt.

On the entry pages, we currently limit annotations to those found in an IUCr publication that also represents the primary citation for a PDB entry. An example of how annotations are disseminated and displayed on PDBe entry pages is shown in Fig. 5 ▸ for PDB entry 5cxt and in Supplementary Video S2. All residues for which annotations are available carry a blue circle in the sequence viewer, whereas bold face is used for residue highlighting in the annotation table. The annotation table, sequence and molecule viewer are all interactively synchronized and a user can click on residues in any of these interfaces to select the subset of relevant annotations. Table 7 ▸ provides a few examples of publications and associated structures for readers to explore.

To support users, we recorded two tutorial videos showcasing how the annotations can be accessed through the PDBe entry pages and via the PDBe API endpoints (Supplementary Videos S2 and S3). For the API endpoints we also included a Jupyter Notebook (PDBe_API_for_text_mined_annotations.ipynb) with examples in the code repository.

## 4. Discussion

Generally, we note that the performance of the underlying model in our system is determined by the training data used during development. As already described in Vollmar *et al.* (2024 ▸), the training data were severely imbalanced, with some entity types represented by very few examples and/or little diversity for the model to learn generalization patterns.

To ensure the implementation of our annotation-tool functions in a production environment, we quantized the model, with an acceptable loss of no more than 0.05 in overall performance metrics, precision, recall and F1 score. This loss of performance allowed us to deploy a much smaller model on standard hardware. Accepting the lower performance affects the entity types ‘mutant’ and ‘residue name number’ differently, with the former already exhibiting lower performance in the full model, which is further reduced due to quantization. Precision measures the accuracy of positive predictions, while recall measures the ability of the model to find all relevant instances. The low precision for ‘mutant’ by the quantized model indicates that the model predicts a large number of false positives for this entity type. However, the recall indicates that the model can correctly identify the majority of ‘mutant’ cases in the benchmarking set.

The overall lower prediction confidence of the model resulted in a number of side effects that defined the limitations of the pipeline:

(i) Entities that consist of multiple words may be predicted as a set of annotations rather than a single one.
(ii) Entity types may be assigned wrongly.
(iii) Entities may be missed by the model entirely.
(iv) False-positive entities are predicted.

The first point can be mitigated by post-processing to fuse neighbouring entities if they are of the same type, which is performed as part of our annotation pipeline. The second issue we addressed through a series of regular expression checks, as described in Sections 2.1.5 and 3.2.

The third issue cannot be addressed through any post-processing steps. A strategy, based on the stochastic nature of these predictive models, could be to repeatedly predict on the same sentences, thus increasing the chance that one of the predictions returns a result. The top five predictions can then be used to determine the most likely result based on a majority count for confidence and entity type. Alternatively, a user-feedback mechanism could be developed through IUCr and PDBe pages to identify missing annotations. The latter would also be beneficial to identify other errors found in annotations. Aside from the model not finding a named entity in the text, an annotation can also be missing because the downstream pipeline was not able to create an association with a sequence in the structure or a UniProt accession. This is usually the case if the authors used a sequence numbering to refer to a residue in the text that does not reflect either of the resources, PDB or UniProt. In such cases, no linking was possible and the annotation will be missing from the IUCr pages, the PDBe relational database, API endpoints and the PDBe entry pages.

The last issue, having a high false-positive rate as we find for ‘mutant’, can in some cases be traced back to having been assigned the wrong entity type and overlaps with issue (iii). Additionally, when developing the predictive model, the definition of ‘mutant’ used was not limited to point mutations, as described in Section 2.1.5. but covered any form of sequence alteration, such as removing anything from a few residues up to whole domains. For example, in the sentence ‘The accumulation of SRP2070Fab molecules by stacking is remarkable and is consistent with the finding that stacking of SRP2070Fab is predominant in known crystal structures of BRIL-fused GPCRs complexed with SRP2070Fab’ from https://doi.org/10.1107/S205979832300311X above, ‘BRIL’ is here not identified as ‘protein’ entity type but as ‘mutant’. This is correct, as in this context BRIL, although being a protein, is also artificially fused to a GPCR and is therefore also of the ‘mutant’ type in the wider sense of our definition. Crucially, we not only check whether an identified entity follows a regular expression pattern for ‘mutant’ or ‘residue name number’, but we also check whether the word span for an entity type can be matched to a residue via SIFTS downstream. If the text span cannot be resolved to match a residue, then the annotation is discarded. In the sentence ‘The gene encoding a Tobacco etch virus (TEV) protease cleavage site and BRIL (Ala1–Leu106) was synthesized and subcloned into pET-28a(+) using BamHI and HindIII’, ‘pET’ is predicted to be of entity type ‘mutant’. We can see that this text span does not match what we would expect for a point mutation or even a deletion, but some of the context in the sentence points towards sequence alterations more generally. However, this annotation comes with a confidence level of ‘ai_score’ 0.46529654, indicating that the model is not very confident in the accuracy of the prediction. Setting a prediction confidence threshold would therefore be another option to filter annotations and exclude false positives.

We successfully processed 76% of the publications of our chosen subset of the IUCr archive for three journals dating back to 2000. The remaining 24% could not be processed for several reasons.

(i) Publications may have been linked to a structure, but there was no mention of specific residues in the text.
(ii) The authors of the publication may not have used a numbering scheme for residues mentioned in the text that could be linked to either the PDB structure or a UniProt accession.
(iii) In some cases, our pipeline failed entirely.

Issues (i) and (ii) are inherent to the specific publication and cannot be fixed through any checks and quality control. Therefore, no annotations will be generated, and although only spot testing was performed for failure cases, these represent the majority of failed publications. We want to stress that our pipeline will not alter the original publication text or fix any sequence-numbering errors. For the third issue, we will investigate the reason for failure for the particular publication and, if possible, improve our pipeline. As we are increasing our expertise in further extending this initial annotation pipeline into a more comprehensive workflow, we will be better positioned to identify the reasons for failure. Improving system robustness will be key for the long-term stability and maintenance of our application. Carefully tracking, through unique identifiers for each publication, and recording intermediate outputs and logging details of failure will provide a start to identify points of failure. For example, when we place external API calls to retrieve additional information, implementing a retry option with a delay will overcome situations where a provider cannot return a response due to high service demand. We already store validation and SIFTS XML files locally to be independent from external services. Creating a look-up in the form of a static file or a database would be another option for long-term storage of information retrieved externally and will only require a new request if data are missing from the local storage. Furthermore, by using unique publication identifiers combined with the recorded errors and issues we can group related issues and continuously improve pipeline robustness to ensure that all publications are processed correctly.

## 5. Conclusions

Here, we present a novel system that enables the association of text mentions of functionally important residues with their corresponding counterparts in a PDB entry and reference UniProt accession. Within the web-based publication environment at the IUCr, these associations are used to highlight functionally important residues in their text context, while a molecular viewer can be opened to view the respective residue in its structural context, supported by experimental quality metrics. We extracted the annotations and shared them with PDBe for indexing and open-sourcing, allowing downstream analysis, further linking on their webpages and dissemination to the wider life-science community.

The main objective for the work presented here is to enable expert structural biology practitioners and non-expert life-science researchers to make informed decisions about scientific findings and conclusions in the context of protein structures and experimental data support. Longer term, the expansion to and incorporation of other experimental databases and resources and structuring the information in a human and machine-readable format is expected to drive novel research and create access to knowledge for non-experts.

## Supplementary Material

Supplementary Video S1. DOI: 10.1107/S2059798326000021/rr5258sup1.mp4

Supplementary Video S2. DOI: 10.1107/S2059798326000021/rr5258sup2.mp4

Supplementary Video S3. DOI: 10.1107/S2059798326000021/rr5258sup3.mp4

## Figures and Tables

**Table 1:** Confidence scores for predicted annotations in an unseen sentence for the full model and the quantized model

Start	End	Text span	Entity type	Confidence, full model	Confidence, quantized model
1	23	N-terminal acetylation	ptm	0.937826	0.9305218
25	39	Nt-acetylation	ptm	0.96235585	0.9603966
57	86	N-terminal acetyltransferases	protein type	0.99854547	0.99813604
88	92	NATs	protein type	0.99888486	0.9987967
162	167	Naa60	protein	0.9994029	0.9992556
180	184	NatF	protein	0.99875224	0.99833703
211	214	NAT	protein type	0.99907637	0.99914896
229	253	multicellular eukaryotes	taxonomy domain	0.97256976	0.9630969
335	349	Nt-acetylation	ptm	0.96585155	0.95354486
397	424	lysine Nɛ-acetyltransferase	protein type	0.9983275	0.9971248
426	429	KAT	protein type	0.99864644	0.9963988
456	467	acetylation	ptm	—	0.9865166
471	477	lysine	residue name	0.99024945	0.99338126
507	525	crystal structures	evidence	0.9975256	0.9964277
529	534	human	species	0.99812895	0.9925248
535	540	Naa60	protein	0.9994475	0.99933916
542	548	hNaa60	protein	0.99933773	0.9990397
550	565	in complex with	protein state	0.99842304	0.99808717
566	583	Acetyl-Coenzyme A	chemical	0.99915624	0.9989887
585	591	Ac-CoA	chemical	0.9992083	0.999053
596	606	Coenzyme A	chemical	0.99916434	0.99896336
608	611	CoA	chemical	0.999223	0.9989459
618	624	hNaa60	protein	0.99931455	0.99901867
645	662	amphipathic helix	structure element	—	0.9946588
645	656	amphipathic	protein state	0.9248927	—
657	662	helix	structure element	0.9796246	—
677	688	GNAT domain	structure element	0.99922186	0.9990057
734	740	hNaa60	protein	0.9993635	0.99923146
750	763	β7-β8 hairpin	structure element	0.99896705	0.9989759
803	809	hNaa60	protein	0.97456926	0.9877958
810	815	1-242	residue range	0.89123815	0.949022
821	827	hNaa60	protein	0.8546437	0.98467755
828	833	1-199	residue range	0.85460573	0.91554123
835	853	crystal structures	evidence	0.9984098	0.9971397
899	905	Phe 34	residue name number	0.99716777	0.99547845
1055	1100	structural comparison and biochemical studies	experimental method	0.9866565	0.993052
1116	1122	Tyr 97	residue name number	0.9976251	0.99692416
1127	1134	His 138	residue name number	0.9974098	0.9968611
1186	1199	non-conserved	protein state	0.9988678	0.9984768
1200	1215	β3-β4 long loop	structure element	0.99922025	0.999139
1250	1256	hNaa60	protein	0.9993338	0.99913245

**Table 2:** Precision determined for the full and quantized model on the respective test set and an independent validation set

Model	Data batch	Strict	Exact	Partial	Type
v2.1, full	Independent validation set	0.78	0.85	0.91	0.85
v2.1, quantized	Independent validation set	0.73	0.80	0.86	0.80

**Table 3:** Recall determined for the full and quantized model on the respective test set and an independent validation set

Model	Data batch	Strict	Exact	Partial	Type
v2.1, full	Independent validation set	0.75	0.82	0.87	0.81
v2.1, quantized	Independent validation set	0.70	0.77	0.83	0.78

**Table 4:** F1 score determined for the full and quantized model on the respective test set and an independent validation set

Model	Data batch	Strict	Exact	Partial	Type
v2.1, full	Independent validation set	0.76	0.83	0.89	0.83
v2.1, quantized	Independent validation set	0.71	0.79	0.85	0.79

**Table 5:** Changes of precision, recall and F1 score for the entity type ‘mutant’ for a full and quantized version of model v2.1 evaluated using SemEval and an independent validation set

Model	Strict	Exact	Partial	Type
Precision, full	0.52	0.52	0.57	0.62
Recall, full	0.81	0.81	0.89	0.97
F1 score, full	0.63	0.63	0.70	0.76
Precision, quantized	0.43	0.43	0.49	0.55
Recall, quantized	0.71	0.71	0.81	0.91
F1 score, quantized	0.54	0.54	0.61	0.69

**Table 6:** Changes of precision, recall and F1 score for the entity type ‘residue name number’ for a full and quantized version of model v2.1 evaluated using SemEval and an independent validation set

Model	Strict	Exact	Partial	Type
Precision, full	0.96	0.96	0.96	0.96
Recall, full	1.00	1.00	1.00	1.00
F1 score, full	0.98	0.98	0.98	0.98
Precision, quantized	0.92	0.92	0.92	0.92
Recall, quantized	0.93	0.93	0.93	0.93
F1 score, quantized	0.93	0.93	0.93	0.93

**Table 7:** Several examples of publications with annotations and associated structures in the PDB

Publication title	PDB entry	URL at IUCr	URL at PDBe	Open access
UHM–ULM interactions in the RBM39–U2AF65 splicing-factor complex	5cxt	https://doi.org/10.1107/S2059798316001248	https://www.ebi.ac.uk/pdbe/entry/pdb/5cxt?activeTab=llm	Yes
Insights into the binding of PARP inhibitors to the catalytic domain of human tankyrase-2	4tk5	https://doi.org/10.1107/S1399004714017660	https://www.ebi.ac.uk/pdbe/entry/pdb/4tk5?activeTab=llm	Yes
Experimental phasing using zinc anomalous scattering	4dt3	https://doi.org/10.1107/S0907444912024420	https://www.ebi.ac.uk/pdbe/entry/pdb/4dt3?activeTab=llm	Yes
Native sulfur/chlorine SAD phasing for serial femtosecond crystallography	4yop	https://doi.org/10.1107/S139900471501857X	https://www.ebi.ac.uk/pdbe/entry/pdb/4yop?activeTab=llm	Yes
Octameric structure of *Staphylococcus aureus* enolase in complex with phosphoenolpyruvate	5bof	https://doi.org/10.1107/S1399004715018830	https://www.ebi.ac.uk/pdbe/entry/pdb/5bof?activeTab=llm	Yes
Sent packing: protein engineering generates a new crystal form of *Pseudomonas aeruginosa* DsbA1 with increased catalytic surface accessibility	4zl7	https://doi.org/10.1107/S1399004715018519	https://www.ebi.ac.uk/pdbe/entry/pdb/4zl7?activeTab=llm	Yes
